# Adjuvant osimertinib treatment in patients with early stage NSCLC (IB-IIIA): pathological pathway adaptations

**DOI:** 10.18632/oncotarget.28210

**Published:** 2022-03-03

**Authors:** Antonio Marchetti, Fiamma Buttitta, Emanuela D’Angelo

**Affiliations:** ^1^Department of Medical, Oral and Biotechnological Sciences, Centre for Advanced Studies and Technology (CAST), University of Chieti, Chieti, Italy

**Keywords:** non-small cell lung cancer, osimertinib, diagnostics, early, survival

## Abstract

Non-small cell lung cancer (NSCLC) is the most common type of lung cancer. Around 30% of patients are diagnosed with early disease and 60% after the tumour has spread to a different part of the body. The earlier NSCLC is diagnosed, the better the chances of prolonging survival. Recent years have seen striking improvements in cancer treatment outcomes through increased use of molecular diagnostics. Therapy decisions are now based on a combination of genetic testing and genetically matched targeted therapies. The positive results obtained with the use of tyrosine kinase inhibitors (TKIs), including osimertinib, in the metastatic disease, coupled with recent data in early stage disease support the importance of molecular testing in this setting. In this overview we discuss factors paramount in pathological pathways to ensure optimal management of early stage NSCLC and also provide an overview of requirements/recommendations. Critical issues in the pre-analytical phases regarding both cytology/biopsy samples and surgically resected tissues are highlighted and solutions are proposed to guarantee accuracy, adequacy and sustainability in the innovative approach to be introduced in clinical practice for NSCLC patients.

## INTRODUCTION

Non-small cell lung cancer (NSCLC) accounts for 85% of all lung malignancies and is still one of the major causes of death from malignant neoplasia in both men and women [1]. In recent years there have been significant advances in the use of targeted therapies for specific molecular alterations. An accurate and detailed histological diagnosis using international guidelines is vital in determining the optimal choice of treatment for individual patients. The 2015 World Health Organization (WHO) classification of lung tumours provided not only a comprehensive guide for surgical resections, but also for small biopsies, where the morphological criteria for the correct diagnosis may not be met due to lack of sufficient material of tissue or cytological samples for molecular evaluation [2, 3].

Establishing a framework for the use of prognostic and predictive markers in defining the therapeutic approach depends on a correct staging of lung cancer at diagnosis [4]. The TNM system, based on the evaluation of three parameters (extension of the primary tumour, lymph node involvement and distant metastases), is universally accepted to determine prognosis and to define the best treatment strategy. In recent years, targeted therapies including inhibitors of the epidermal growth factor receptor (EGFR TKIs), and anaplastic lymphoma kinase (ALK) have been developed. The presence of activating EGFR mutations in NSCLC represents the most important predictor for adopting targeted therapy with first (gefitinib, erlotinib), second (afatinib) or third generation (osimertinib) EGFR TKIs, shown to offer significant clinical benefits to patients over standard chemotherapy [5–9] National and international guidelines recommend that EGFR mutational status is established to determine the optimal therapeutic strategy in individual patients with advanced NSCLC as well as to improve understanding of the tumour biology on disease progression and acquired resistance [10–12].

Results of a Phase 3 study in patients with stage IB to IIIA resected EGFR mutation–positive NSCLC, showed that disease-free survival (DFS) was significantly longer among those who received adjuvant therapy with osimertinib compared with those receiving placebo [13]. In light of these results, modifications/improvements to pathological pathways (including mutational analyses of the EGFR receptor) are required to ensure adequate diagnostic work-up in an increasing population of patients with NSCLC.

## TYROSINE KINASE RECEPTOR INHIBITORS (TKIS) IN EARLY STAGE NSCLC

Advances in precision medicine leading to the development of molecular therapies were a major step forward in the management of NSCLC and drugs aimed at specific molecular targets are now fundamental in treating advanced NSCLC. In recent years the focus of the scientific community has been on their use in stage IB-IIIA disease, to increase efficacy alone and in combination with adjuvant chemotherapy as well as improving overall tolerability. TKIs are used as first-line treatment in patients with locally advanced (stage IIIB - that cannot go through chemo-radiotherapy treatment) or metastatic (stage IV) NSCLC harbouring sensitizing EGFR mutations – key predictive markers for patient selection. Currently, the first-line standard of care treatment is osimertinib, a third-generation, irreversible EGFR-TKI that selectively inhibits both EGFR-sensitizing and EGFR T790M resistance mutations. The phase 3 FLAURA trial assessed the efficacy and safety of osimertinib in patients with previously untreated EGFR mutation–positive advanced NSCLC compared with standard EGFR-TKIs, gefitinib or erlotinib. Osimertinib treatment resulted in significantly longer progression free survival (PFS) compared to previous EGFR-TKIs. Median PFS was 18.9 months (versus 10.2 months), overall response rate 80% (versus 76%) and median duration of response was 17.6 months (versus 9.6 months) with a 54% lower risk of disease progression [9]. A preplanned subgroup analysis with CNS progression-free survival as the primary objective was conducted in patients with measurable and/or nonmeasurable CNS lesions on baseline brain scan showed that the probability of experiencing a CNS progression event was consistently lower with osimertinib versus standard EGFR-TKIs [14] This represents a major advantage as the presence of brain metastases is increased in EGFR-mutation positive NSCLC with a incidence at diagnosis of 25/30% and a 15–20% risk of CNS progression during first generation EGFR TKIs treatment [15]. Importantly in the FLAURA study median overall survival (OS) was 6.8 months longer in the osimertinib group than in the comparator (38.6 versus 31.8 months), with a 20% lower risk of death, even in the presence of crossover from the comparator group to the osimertinib group [16]. Furthermore, at 36 months, three times as many patients were continuing to receive the assigned trial drug in the osimertinib group than in the comparator group [16]. Results from a follow-on patient-reported outcome study using the FLAURA cohort showed improvements in the osimertinib arm that were statistically significantly greater than in the erlotinib/gefitinib arm for emotional functioning (8.79 vs. 4.91; *p* = 0.004) and social functioning (7.66 vs. 1.74; *p* < 0.001). Cognitive functioning remained stable in the osimertinib arm but deteriorated in the erlotinib/gefitinib arm (0.03 vs. 3.91; *p* = 0.005). Improvements in global health status/quality of life (QoL) and functional scores from baseline to randomized treatment discontinuation were seen in both treatment arms. None of the mean changes reached the 10-point improvement threshold for clinical relevance [17].

Approximately 30% of patients with NSCLC present at an early disease stage (stage I, II and IIIA). In these patients the treatment of choice is surgical resection, with the possibility of complete removal of the tumour mass with a curative intent. Overall, mean 5-year OS rates for early stage NSCLC range from 84% for stage IA to 36% for stage IIIA [4]. However, relapse rates after surgery remain high, irrespective of the use of postoperative chemotherapy, and additional therapeutic approaches are required. Adjuvant cisplatin-based chemotherapy is currently recommended for stage II and IIIA disease, after complete resection, and may also be considered for patients with stage IB cancer >4 cm [10, 12]. In patients with preoperative lymph node (N2) metastasis (considered to be non-resectable), a combined chemo-radiotherapy approach can be adopted, which can be followed with durvalumab consolidation therapy in patients with disease control. Chemotherapy treatment followed by surgery also represents a valid option, with preference for surgery in patients in whom complete resection via lobectomy is possible [10]. While the efficacy of adjuvant chemotherapy has been demonstrated — clinical studies and related meta-analyses show a significant improvement in OS (5% at 5 years) in patients with early stage NSCLC who received adjuvant chemotherapy — further treatment is required to fulfil the significant unmet clinical need [18, 19].

The efficacy of adjuvant TKIs in patients with EGFR mutation-positive early stage NSCLC has been investigated in clinical studies. The EVAN study compared adjuvant erlotinib therapy with standard vinorelbine and cisplatin administered for 2 years, in patients with EGFR-mutant stage IIIA NSCLC who had undergone resection. DFS at 2 years was 81.4% in the erlotinib group and 44.6% in the chemotherapy group [20]. The ADJUVANT study in patients with EGFR-mutant stage II or IIIA NSCLC who received either adjuvant therapy with gefitinib or chemotherapy with vinorelbine and cisplatin after surgery for 2 years of treatment, showed an improvement in median DFS of 28.7 months with gefitinib versus 18 months with chemotherapy [21]. These studies used first-generation EGFR TKIs, such as gefitinib and erlotinib, however the current standard of care in EGFR-mutated advanced NSCLC is the third generation TKI osimertinib. ADAURA, a randomized, double-blind, phase 3 trial evaluated the efficacy and safety of treatment with adjuvant osimertinib compared to placebo (PBO) in patients with stage IB-IIIA EGFR mutation positive tumours. In this study adjuvant chemotherapy was allowed prior to randomization. Overall, 682 patients were enrolled (339 treated with osimertinib, 343 with placebo) for whom the main clinical-pathological and molecular characteristics (stage, sex, type of mutation) were examined. The primary endpoint of ADAURA trial was DFS in stage II-IIIA patients: the hazard ratio (HR) was 0.17 (99.06% confidence interval [CI], 0.11–0.26); the 2-year DFS was 90% for osimertinib treatment and 44% for PBO treatment. In the stage IB-IIIA overall population, the HR for DFS was 0.20 (99.12% CI, 0.14–0.30) and the two-year DFS was 89% after treatment with osimertinib and 52% after treatment with PBO [13, 22]. The safety and tolerability of osimertinib was consistent with previous Osimertinib studies in EGFR-mutation positive metastatic NSCLC patients [13]. Adjuvant osimertinib is the first targeted agent to show a statistically significant and clinically meaningful improvement in DFS in patients with stage IB/II/IIIA EGFR-mutated NSCLC after complete resection and adjuvant chemotherapy (when indicated). This regimen may become the standard of care in the adjuvant setting for patients with stage IB to IIIA NSCLC. These encouraging results open up significant prospects for treating patients with targeted drugs in earlier stages of NSCLC.

## NEW SCENARIO IN THE DIAGNOSTIC PROCESS FOR EARLY-STAGE NSCLC

Despite the advancement in technology and cancer research, 57% of lung cancer patients are diagnosed only after the tumour has metastasized to a different location [23]. Detection at Stage I or II for NSCLC can offer good prognosis and it is evident that the earlier lung cancer is diagnosed, the better the prognosis. However, in the early stages of tumour pathology, problems related to clinicopathological aspects, in particular the pre-analytical phase, may affect a correct diagnosis. These critical diagnostic issues are different from those in patients with more advanced clinical stage tumours and as such must be specifically managed before being introduced into clinical practice. It is necessary to first examine the fundamental points that need to be addressed in order to improve the pathological path for future clinical and therapeutic developments.

It is known that the pre-analytical phase is essential for the success of all subsequent phases of morphological, immunophenotypical and molecular characterization of the tumour that in turn represent the main juncture in the therapeutic process and patient management. Nevertheless, the importance of this phase is often underestimated, sometimes compromising the entire diagnostic process. Biological material used for molecular analyses in diagnosing early stages of the disease (IB-IIIA) can be cytological/biopsy samples taken for diagnostic purposes before surgery as well as surgically resected tissue samples. While the diagnostic process for cytological/biopsy samples is usually conducted rapidly and accurately this is not always the case for surgical resections when analyses are not conducted with the same rigor and speed as biopsy samples.

## PRE-ANALYTICAL FLOWS: CRITICAL ISSUES AND CORRECTIVE ACTION

Optimizing clinicopathological pathways for patients with early operable cancer requires a targeted and precise approach for both cytological/biopsy material and for surgically resected samples. In the management of both types of samples there are critical aspects that require specific solutions to ensure an accurate morphological and molecular diagnosis on cytological/biopsy material before surgery or if this is not possible, on resected tissue. Although there has been a rapid increase in clinically relevant biomarkers for advanced-stage NSCLC the lack of sufficient tissue samples for molecular analysis is a major drawback. While surgical and biopsy samples remain the gold standard for molecular purpose, in real-world clinical practice obtaining large tissue specimens is not always possible and, in most cases, pathologists have to make do with small tissue samples such as endoscopic biopsies and cytological materials and not all samples are of sufficient quality for analysis [24, 25] (Table 1).

**Table 1:** Critical aspects of the pre-analytical phase for small samples (biopsy/cytology material)

**Quantity**	Quantity may not always be sufficient for an accurate differential diagnosis of non-squamous carcinoma versus other histotypes of lung cancer, and/or for an in-depth molecular analysis.
**Quality**	Biological material may be of poor quality due to the presence of coarctated and/or necrotic areas that do not permit the immunophenotypic and molecular investigations necessary for accurate diagnosis and treatment.
**Representativity**	Given the possible heterogeneity of some cancers, cytological/ biopsy micro-sampling may not be representative of the overall lesion, in that the tumours can be mixed and have multiple components, some of which may escape analysis.

**Figure 1:**
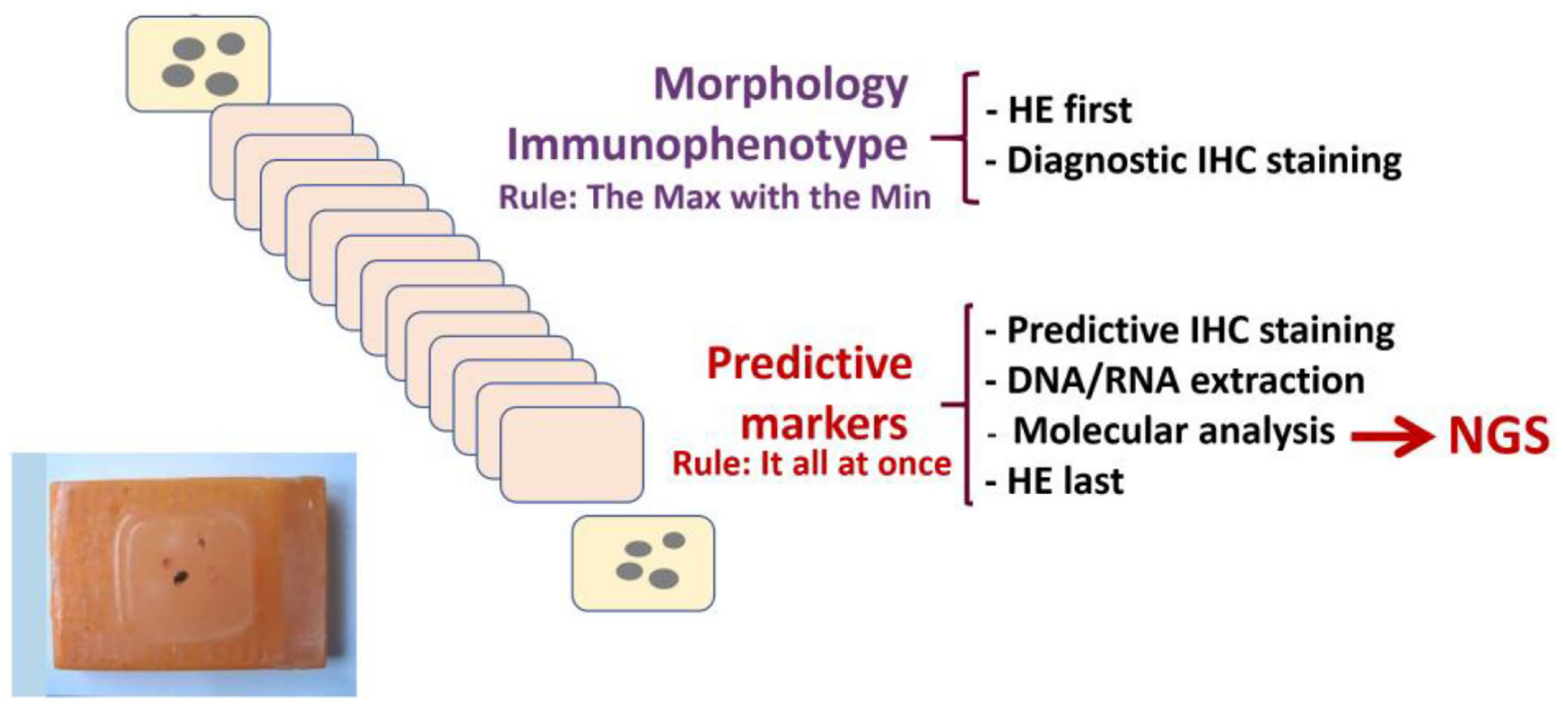
Optimization of the pre-analytical workflow for paraffin-embedded biopsy/cytology material. A series of sections (5 microns) are taken from a paraffin block in a single cutting session. Morphological and immunophenotypical characterization should be carried using the minimal amount of tissue sections to conserve material for further analyses. Additional sections are required to test the tumour for predictive biomarkers which involves immunohistochemical staining to assess the expression of predictive protein markers (PD-L1, ALK-1, ROS1, etc) and DNA-RNA extraction from the remaining sections for comprehensive genomic analysis by next generation sequencing. In order to produce a comprehensive report, it is important to evaluate all biomarkers concomitantly. Abbreviations: HE: haematoxylin and eosin; IHC: immunohistochemistry, NGS: Next generation sequencing.

Given the small amount of cytological/biopsy material, the pathologist must do everything to preserve and make good use of the limited cells/tissue available and bear in mind that when sections are repeatedly cut from the paraffin block, over time material is lost which may be important for the characterization of the tumour. Material is lost each time the block is removed and repositioned in the microtome requiring numerous cuts to bring the block back to level. In our hands, the best way to preserve and optimize a small sample is to cut several (as many as required) sections at one time to carry immediately all the morphological and molecular analyses and to keep the rest of the block for future investigations (Figure 1). If it is not possible to obtain cytological/biopsy material or despite the presence of the material, it is not possible to make a complete morpho-molecular analysis and the patient is subjected to exploratory surgery, the pathologist can use the resected specimen for diagnostic purposes [26]. It should be noted that resected tissue must be managed appropriately – although a larger amount of material is available to the pathologist compared with that from a biopsy, there are other issues that need to be managed and prevented.

## SURGICALLY RESECTED TISSUES: OPTIMIZING PROCEDURES

A key step in diagnosis is an accurate analysis of surgically resected material – it is therefore vital that correct procedures are followed by adopting two main recommendations (Table 2).

**Table 2:** Recommendations for an accurate pre-analytical process of resected tissues

**First recommendation**	Reduce as much as possible the time after the removal of the tissue and before fixation (of cold ischemia) to 15–30 minutes.
**Second recommendation**	Adopt specific fixation and inclusion strategies for the resected tissue to meet qualitative standards and the optimal pathways of biopsies.

The proposed recommendations are fundamental not only to improve the quality of histopathological/immunophenotypic analyses but also to accurately characterize the tumour using molecular investigations, particularly whith the advent and diffusion in pathology labs of the Next Generation Sequencing (NGS) technology [27, 28].

In the context of neoplastic pulmonary pathology, tissue samples can come from a range of surgical interventions:

- Nodulectomy
- Atypical lung resection
- Lobectomy
- Pneumonectomy

For all these types of tissue samples, fixation of the surgically resected material must be performed properly, as the quality of subsequent immunophenotypic and molecular investigations depends on how accurately the process is carried out. Formalin (10% buffered formaldehyde), used for fixing the tissue sample, penetrates into the tissue slowly (about 1 mm per hour). In certain samples, neoplastic tissue is deeply located and therefore it takes longer for the fixative to penetrate into the tumour tissue — for example, for a tumour that is 2 cm away from the sample surface, it may take around 20 hours for the fixative agent to reach the neoplastic tissue. In order to perform correct tissue fixation, two key elements should be considered as reported in Table 3. In particular, allowing adequate fixation time ensures that molecular tests of good quality can be performed on properly fixed tissue.

**Table 3:** Recommendations for accurate fixation procedure for resected material

**First recommendation**	Receiving the surgical tissue sample in a ‘fresh” state’ – this means that a series of cuts can be made facilitating the rapid and effective penetration of formalin into the areas of interest.
**Second recommendation**	Allow adequate time for fixation: fixation times of between 12 and 48 hours are suggested, preferably around 24 hours. Fixation times of <8 hours or >72 hours considerably affect the immunoreactivity of tissue and the quality of the nucleic acids extracted for molecular tests.

It is vital to go through the various phases of fixation, inclusion, cutting and staining of resected surgical specimens using optimal predefined timings [25, 27]. The objective is to adhere as far as is technically possible to timings of these phases with those of the preparation of biopsy samples, which currently have priority. Specifically, in clinical practice it is necessary to reduce the pathological reporting time for resected samples from the current 2–3 weeks to one working week. In conclusion, for surgically resected tissue samples it is necessary to reorganize the pathological pathway to correctly define all the phases. The definition and implementation of optimal timing and methods for carrying out the various pre-analytical phases can allow adequate management of the diagnostic-staging path of the patient with NSCLC.

## OPTIMIZATION OF THE MANAGEMENT OF EARLY STAGE NSCLC

Over the past decades, we have seen impressive improvements in cancer treatment outcomes through the combined use of molecular diagnostics and targeted therapies. Previously cancer treatments were linked to specific histological types – different drugs for different cancers – but now, in an increasing number of situations, treatment decisions are based on a combination of genetic testing and genetically matching targeted therapies. In this paper we discuss the factors, in particular accuracy, completeness and timeliness, that are vital in the pathological pathway in order to ensure optimal management of early stage NSCLC. In an ideal world, an oncologist would be in a position to specify the optimal targeted therapeutic strategy, for example including adjuvant treatment with osimertinib, for a patient with newly diagnosed EGFRm NSCLC as all the information on genetic mutations in the tumour would be available at the outset. For this to happen it requires a paradigm shift in the diagnostic workup in general and the for the pathologist in particular. In everyday clinical practice, this would mean that at the time of the clinical diagnosis, it would be the pathologist who makes the request and starts the procedure referred to as molecular profiling or mutation profiling. As the importance of understanding the genetic characteristics of a lung tumour cell increases, pathologists and pulmonologists encourage that ‘reflex testing’ for genetic mutations and biomarkers is conducted. Reflex testing involves performing testing for clinically relevant lung cancer mutations at the same time that diagnostic testing is carried out, irrespective of the patient’s tumour staging – in order to speed up the overall process. Tests carried out in this way complete the histological report and the diagnostic process and reduce the time required to treat the patient in the most appropriate way. Although the benefits of targeted therapy are well documented many eligible patients are still not receiving recommended therapies. Molecular testing of lung cancer is important to ensure that patients receive optimal treatment, however data show that to date limited numbers of pulmonologists and pathologists implement reflex testing of NSCLC patients in their practice. In one survey most respondents who test and treat patients believe that less than 50% of patients with lung cancer in their country receive molecular testing [26, 29]. Barriers cited included cost, access, quality, turnaround time, and lack of awareness with cost the most important barrier identified. There is a need for improved collaboration between members of the multidisciplinary team to work together to achieve the common goal of initiating an appropriate lung cancer treatment plan as soon as possible. One way forward is the instigation of Molecular Tumour Boards (MTBs) which help to ‘translate increasingly complex genetic information into patient-centred clinical decisions’ and are considered ‘critical to close the growing gap between clinical practice and technological potential in cancer care’ [30]. Although this new paradigm brings with it economic consequences, costs of reflex tests may be included in the clinical-diagnostic pathways or Diagnosis Related Groups (DRG) – a patient classification system that standardizes prospective payment to hospitals and encourages cost containment initiatives [31]. In general, a DRG payment covers all charges associated with an inpatient stay from the time of admission to discharge. Paradoxically, this approach may mean in the long-term a greater appropriateness of examination requests and even knock-on savings.

Finally, specific training for pathologists will be required to improve the diagnostic workflow and meet the new treatment needs for patients with early stage NSCLC. The changes in the therapeutic landscape in recent years have highlighted the need for a network of professional relationships within interdisciplinary working groups. Interactivity in working groups is vital. It promotes training, discussion and appropriateness in patient management that is based on specific recommendations and guidelines with the overall objective of obtaining the best results in terms of both survival and quality of life [31].
